# Developing and testing a nursing home end -of -life care chart audit tool

**DOI:** 10.1186/s12904-018-0301-9

**Published:** 2018-03-15

**Authors:** Genevieve N. Thompson, Susan E. McClement, Nina Labun, Kathleen Klaasen

**Affiliations:** 1 College of Nursing, Rady Faculty of Health Sciences, University of Manitoba, Winnipeg, MB R3T 2N2 Canada; 2 Donwood Manor, 171 Donwood Dr, Winnipeg, MB R2G 0V9 Canada; 3 Winnipeg Regional Health Authority, 4th floor, 650 Main St, Winnipeg, MB R3B 1E2 Canada

**Keywords:** Palliative care, Nursing home, Quality of care, Audit tool, Tool development, Tool validation, Focus groups

## Abstract

**Background:**

Nursing home (NH) administrators need tools to measure the effectiveness of care delivered at the end of life so that they have objective data on which to evaluate current practices, and identify areas of resident care in need of improvement.

**Methods:**

A three-phase mixed methods study was used to develop and test an empirically derived chart audit tool aimed at assessing the care delivered along the entire dying trajectory.

**Results:**

The Auditing Care at the End of Life (ACE) instrument contains 27 questions captured across 6 domains, which are indicative of quality end-of-life care for nursing home residents.

**Conclusions:**

By developing a brief chart audit tool that captures best practices derived from expert consensus and the research literature, NH facilities will be equipped with one means for monitoring and assessing the care delivered to dying residents.

## Background

Nursing homes (NHs) have increasingly become the final residence for a growing cohort of individuals living with a variety of chronic life-limiting conditions. The acuity and medical complexity of residents entering NHs is increasing; current estimates indicate that the average resident dies within 2 years after admission [1] and 81% of those admitted to NHs will die there [2–4]. Given this reality, care providers in NHs face the challenge and responsibility of providing residents with quality end-of-life care. NH administrators need tools to measure the effectiveness of care delivered at the end of life so that they have objective data on which to evaluate current practices, and identify areas of resident care in need of improvement. One way of obtaining such data is through a chart audit, since these audits are designed to improve patient care by identifying gaps in care through the systematic collection and analysis of data [5]. Chart audits are routinely conducted in many areas of health care [6–8].

Though a limited number of studies have been conducted using chart audits to evaluate the quality of palliative care in hospital [9], primary care [10, 11], and hospice/home care [12], studies using audits in the NH setting are limited. A review of the literature identified only two research studies that have conducted a chart audit of the care of the dying in NH [12, 13]. However, both focused only on the immediate time before death, and neither specifically focused on developing a chart audit tool that was designed for routine use in clinical practice. Since excellence in palliative/end-of-life care espouses attending to the physical, psychosocial, and spiritual needs of the dying longer than the imminently dying phase [14], a thorough understanding of the care a resident receives for their entire dying phase is essential to understanding the quality of care provided during this time.

In order to address the limitations of the studies conducted to date and to construct a clinically relevant audit tool, the overarching goal of this study was to develop and test an empirically derived chart audit tool aimed at assessing the care delivered along the entire dying trajectory. Specific objectives for this study were to: (1) identify items that are indicators of quality end-of-life care important for inclusion on a chart audit tool; (2) develop, validate, and refine the NH resident end of life audit tool informed by objective one; and (3) evaluate the acceptability and utility of the tool in practice.

## Methods

A three-phase mixed methods study was conducted to address the study objectives. Using the procedures outlined by Steiner and Norman [15] for the development and testing of health measurement scales, the research team engaged several processes to meet the objectives: 1) a critical review of existing literature; 2) item generation; 3) selection of items and assessment of validity; 4) field trials; and 5) generation of a refined instrument. This study received ethical approval from the University of Manitoba Education/Nursing Research Ethics board.

### Phase I

The purpose of phase one of the study was to generate a list of possible end of life data elements for a chart audit tool that would capture best practices in the provision of care to dying residents. Drawing on the expertise of our research team, along with a critical review of the literature on best practices in end-of-life care for residents dying in NHs, the areas described in a framework for a good death by Bosek and colleagues [16] were used to determine broad headings under which items to include in the chart audit would be placed. These headings included pain and symptom management, clear decision making, preparation for death, and affirmation of the whole person. Discussion concerning the time frame in which to evaluate end-of-life care was guided by our review of the literature. The team also reviewed existing audit tools currently used within the local regional health authority in order to have a template for the range of items, the manner in which questions can be formatted, and the types of data that are best collected using an audit tool.

### Phase I analysis

Following the methodology proposed by Gearing and colleagues [17], the information collected in phase one was collectively coded, categorized and synthesized to ensure items generated for inclusion on the draft audit tool were: 1) representative of the experience of a good death in the NH setting, common occurrences, and areas of significant concern,; 2) grouped under the broad conceptual domains that emerged in the review of the literature,; and 3) included both quantitative and qualitative items in order to capture the care delivered in the last month and week of life. For example, simple yes/no questions were used to indicate the presence of a specific symptom, and larger free text areas were provided to capture the type of psychosocial care provided to family members. At this point, by consensus of the research team, and guided by the review of the literature, it was decided that the audit tool would evaluate the care the resident received in their last month of life. The audit tool was formatted following the advice of Allison [5] and Banks [18] who stipulate that a well-designed audit tool is designed to promote accuracy of data capture and to limit the likelihood of missing data.

### Phase II

Phase two saw the development, validation, and refinement of the chart audit tool. A convenience sample of key decision makers, gerontological and palliative care clinicians involved in providing care to NH residents (either direct clinical care or administrative services) were recruited to participate in a focus group in order to provide feedback regarding the clarity, content, and scope of the audit tool.

The administrative staff at a large urban regional health authority long-term care program in central Canada, sent a letter of invitation to potential focus group participants via email. The invitation instructed them to email the study’s research assistant directly to indicate their interest in taking part. The research assistant determined if the participant met eligibility criteria, clarified any questions regarding the study, and provided the details of the focus group such as date, time and location. The research assistant also provided participants with a copy of the draft chart audit tool to review prior to the focus group. Focus group participants signed a consent form and completed a demographic questionnaire prior to the start of the discussion.

During the focus groups, participants were invited to share any insights and concerns they had about the audit tool. Each question on the audit tool was systematically discussed to assess its clarity, relevance, and importance. The focus group was facilitated by the first author while detailed notes of the discussion were recorded by the research assistant. The focus group lasted 45 min. The feedback obtained from participants regarding the clarity, relevance, and importance of the items along with any insights or concerns raised during the focus group was used to revise the tool. The final version of the tool was vetted by the research team prior to pilot testing, to review the items, and formatting of the draft audit tool.

### Phase II analysis

In the course of conducting focus groups, critical appraisal of the draft audit tool was completed by gerontological and palliative care health care providers and administrators (*n* = 12). Focus group participants were on average 47 years old (median 49 years), with 23.95 years of experience in their profession (range 8–37 years) and 11.95 years working in long-term care (range 2–26 years). The group was comprised of registered nurses (*n* = 5), clinical nurse specialists (*n* = 2), a social worker (*n* = 1), a pharmacist (*n* = 1) and long-term care administrators (*n* = 3). All items on the audit tool were highly endorsed by the group, including the assessment period (i.e. last month of life). Minor revisions to the instrument questions were made to correct terminology (e.g., advance care planning levels of care) and minor corrections to the formatting of the tool were made based on comments derived from the research team.

### Phase III

**Fig. 1:**
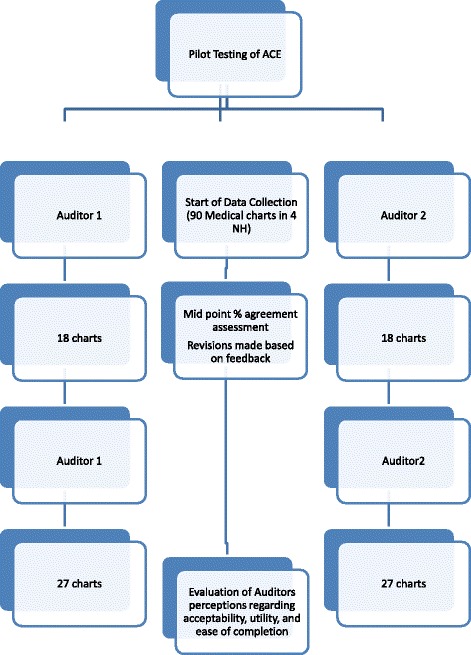
Phase III protocol

In the final phase of this study, a brief trial of the audit tool was conducted in order to assess the reliability, time to complete, and the acceptability and utility of the instrument (See Fig. 1). Four NH facilities participated in the testing of the tool from which a random sample of 20% (*n* = 90) of their deceased residents’ charts from between January and December 2012 were audited; a sample size recommended for chart audits [19]. Two chart auditors with clinical experience in palliative care/gerontology were trained in the use of the audit tool for this study. The auditors independently extracted data from four of the same charts derived from the overall sample, in order to assess for ambiguous or confusing information on the audit tool. The abstracted data was compared for evidence of agreement on major variables. Every instance of variance was reviewed, using information in the original chart as the reference for further discussion [20]. The main challenge identified was that assessing end of life symptom management in the last month of life required too much data abstraction from the chart, contributing to an unacceptably long completion time of the tool (on average 73 min). Based on discussions between the auditors and research team, the period of symptom management assessment was changed to focus on the last week of life, while the remainder of the items on the tool continued to assess care provided in the last month of life.

Once consensus was reached between the chart auditors and research team as to the clarity of the items on the audit tool, the auditors independently completed 18 of the same charts in order to assess inter-rater reliability. Once those charts were analyzed, each auditor independently abstracted a sub-sample of the remaining charts (*n* = 58). Each auditor kept a journal detailing any difficulties they encountered while using the tool, suggestions for refining the tool, and the general ease of use of the tool.

### Phase III analysis

The reliability of the chart audit tool was assessed through the evaluation of percentage agreement. This method was chosen since calculation of inter-rater reliability using Cohen’s kappa (Kappa) ratings is dependent upon the distribution of the data; when data are skewed, for example by raters selecting the same response categories, reliability measures are low [21]. The percentage agreement was calculated from the number of ratings with agreement on the 18 charts coded at mid-point. For each item on the instrument, a percentage agreement of 80% or greater was considered to indicate reliability. The average time to complete a chart audit was computed based on the total time required to extract data from each chart and the number of charts reviewed. The journal notes kept by the auditors and from the meetings with the research team were coded for major themes using content analysis and constant comparative techniques to assess the acceptability, utility, and ease of using the chart audit tool [22].

## Results

The final version of the instrument was named the Auditing Care at the End of Life (ACE) instrument, and it contains 27 questions captured across 6 domains (Table 1). These domains were conceptualized based on the review of the literature (occurrence and management of symptoms; acknowledgment of and preparation for dying; evidence of advance-care planning; attending to spiritual health and cultural aspects of resident care) and to provide context for the care being audited (resident demographics, the factors surrounding the death [e.g. place of death, cause of death, hospitalizations]). Questions were developed for each of these six domains that captured best practices in the delivery of end-of-life care.

**Table 1:** Example ACE tool domains and questions

Domain 1: Demographics
● Date of birth ● Date of death ● Gender ● Length of NH stay (in months)
Domain 2: Situation around death
● Indication on health record that death was expected? (YES/NO) ● Place of death (NH/Chronic Care/Acute Care/Palliative Care Unit) ● Was the resident transfered to acute care in their last month of life? (YES/NO)
Domain 3: Clear decision making
● Was there an Advance Care Plan? (YES/NO) ● Any changes to the Advance Care Plan made at last review? (YES/NO) ● Was there a Health Care Directive (YES/NO)
Domain 4: Preparation for death
● Is there evidence in the progress notes that staff recognized changes in the resident's condition that acknowledged that end of life was near? (YES/NO) ● Were there changes or adjustments made to the resident's physician/NP orders in the last month of life? (YES/NO) ● Were there any medication changes made in the last week of life? (YES/NO) ● Is there evidence in the progress notes that psychosocial support was provided to family members or friends during the dying experience? (YES/NO) ● Is there evidence in the progress notes of communication with family or friends about end-oflife care? (YES/NO)
Domain 5: Spiritual health and cultural aspects of care
● Evidence of resident’s or family wishes regarding rites and rituals, or spiritual considerations acted upon (e.g., minister/pastor called, last rites administered)? (YES/NO) ● Resident's spiritual health preferences documented? (YES/NO)
Domain 6: Symptoms and symptom management through the death
● Is there evidence that Pain was assessed?(YES/NO) ● If a symptom (physical or psychological) is present, describe the managment. ● Personal Care/Comfort Provided in last week of life (e.g.: bathing, mouth care, positioning; incontinence care)?(YES/NO) ● Were consults made for other resources (e.g. Social Work, Volunteers, Clinical Nurse Specialists, Speech Language Pathologist)? (YES/NO)

In the pilot testing of the ACE instrument, the average time to complete an audit of one resident chart was 28.2 min (range: 15–60 min). The percentage agreement by the raters with the 27 questions on the 18 charts ranged from 61 to 100% (see Table 2). Overall, the two auditors found the instrument easy to use however, the question on symptom management was the most “problematic”. For example, the auditors noted that it was frequently difficult to read the handwriting in the chart, that often a standard tool for pain assessment was not used (though it was noted in the progress notes that the resident was in pain), and that rarely was it noted that follow up or evaluation regarding the effectiveness of an intervention occurred (e.g. administrating pain medication).

**Table 2:** Percentage agreements between auditors on the ACE items

Instrument item #	Number of scores in agreement (*n* = 18)	Percent agreement	Description of question on ACE
6	18	100	Indication on health record death was expected
9	18	100	Place of death
10	18	100	Was the resident transferred to acute care (ED) in the last month of life?
11	18	100	Was there a Health Care Directive?
12	18	100	Was there an Advance Care Plan (ACP)?
12a	17	94	Goals of care chosen
14	15	83	Any changes made to the last ACP?
15	18	100	Is there evidence that EOL was near?
16	16	88	Were there changes to resident’s orders in the last month?
17	18	100	Were there medication changes in the last week?
18	15	83	Is there evidence of communication with family?
19	17	94	Is there evidence that psychosocial support was provided to the family?
20	16	88	Resident’s spiritual health preferences documented?
21	15	83	Evidence of resident’s or family wishes regarding rites and rituals, spiritual considerations acted upon?
22	17	94	Is there evidence that pain was assessed?
24a	13	71	Pain present?
24b	18	100	Nausea present?
24c	16	88	Vomiting present?
24d	18	100	Constipation present?
24e	15	83	Diarrhea present?
24f	17	94	Dysphagia present?
24 g	15	83	Dyspnea present?
24 h	15	83	Respiratory congestion?
24i	14	77	Cough present?
24j	13	72	Dry mouth present?
24 k	11	61	Fever present?
24 l	13	72	Skin breakdown present?
24 m	17	94	UTI?
24n	17	94	Edema present?
24o	18	100	Seizures present
24p	13	72	Other symptom issues?
24q	17	94	Depression present?
24r	16	88	Anxiety present
24 s	18	100	Agitation present?
24 t	14	77	Delirium present?
24u	13	72	Other psychosocial symptoms present?
25a	16	88	Was mouth care provided in the last week of life?
25b	14	77	Was bathing provided in the last week of life?
25c	16	88	Was incontinence care provided in the last week of life?
25d	17	94	Was positioning provided in the last week of life?
26	17	94	Was the Regional Health End-of-Life Toolkit used?
27a	18	100	Was a consult made for the Regional Palliative care program?
27b	18	100	Was a consult made for other MD or NP services?
27c	18	100	Was a consult made to the site CNS?
27d	18	100	Was a consult made to the Hospice Palliative care volunteer program?
27e	18	100	Was a consult made to the Facility Volunteers?
27f	17	94	Was a consult made to the Speech Language Pathologist?
27 g	17	94	Was a consult made to the Spiritual Health Practitioner?
27 h	16	88	Was a consult made to the Registered Dietitian?
27i	18	100	Was a consult made to the Regional NH CNS?
27j	14	77	Was a consult made to the Therapeutic Recreation?
27 k	18	100	Was a consult made to the Respiratory Therapist?
27 l	18	100	Was a consult made to the Pharmacist?
27 m	16	88	Was a consult made to Social Work?
27n	12	66	Was a consult made to the OT/PT/Rehab services?
27o	18	100	Was a consult made to the manager of food services?
27p	14	77	Were other consults made?
27q	18	100	Was a consult made to the Regional ethics committee?

*CNS* Clinical Nurse Specialist, *NP* Nurse Practitioner

## Discussion

To our knowledge, the ACE instrument is the first audit tool that has been developed to assess the domains of end-of-life care in NH residents and specifically designed to be used in clinical practice. The broad scope and detail covered by the instrument will allow administrators to clearly identify and measure the quality of care delivered to NH residents in their last month of life. This instrument was developed based on an extensive review of the literature on quality care of dying NH residents and validated through a rigorous process.

In the development of the instrument, as well as the testing of the face and content validity using focus group participants with considerable expertise and experience in long-term care, lends credibility to the tool. Further credibility of the instrument was established through pilot testing, where the auditors identified the instrument was easy to use and was completed in a relatively short time. The assessment of the inter-rater reliability showed high percentages of agreement between the two auditors for the majority of the questions on the instrument. Where discrepancies occurred, these were most frequently due to the auditors being required to make a judgment regarding the intent of the note documented. For example, often hydromorphone was documented as being given to the resident in the final days for “comfort” however, it was unknown if this was due to the resident experiencing pain, or for other undocumented reasons. Similarly, when the documentation included having given a family “up-dates,” it was unknown if this qualified as having an end-of-life discussion or if information was simply being provided to the family. Further training of future users of the tool and the development of a manual to guide auditors in its use, would assist in resolving these issues.

While a detailed discussion of the specific findings using the ACE tool is beyond the scope of this paper, several observations made by the auditors deserve comment. First, that in many instances, no standardized tool for pain assessment could be found in the resident’s chart is troubling, but not surprising given the breadth of research documenting the inadequacies in pain assessment and management in this setting [23–27]. In addition, the evaluation of the effectiveness of an intervention and reassessment of pain are critical nursing activities, yet several authors note limitations in this regard [28, 29]. The lack of documentation that was noted by the auditors points to potential problems in the nursing documentation process, something that has been noted by other scholars [30, 31]. It also demonstrates that NH administrators need to reiterating to nursing staff, the importance of documenting the care they provide to the resident and their family. As the adage goes, if it is not documented, we have to assume it has not been done [32].

Some limitations with the instrument warrant comment. There may be some level of subjectivity associated with auditing charts [33], yet specific steps to limit subjectivity were taken as auditors assessed the care documented in the charts. For example, the tool has been developed to record occurrences, or lack thereof, of care; not to evaluate the care itself. Having auditors record whether there is documentation in the record that care has occurred or not and having them provide details surrounding the care delivered, eliminates the auditors need to make judgments regarding the acceptability of the care delivered. However, this then will require the individual who receives the chart audit reports, to then interpret and make a judgment as to the acceptability of the quality of care.

## Conclusion

Auditing the care provided in the resident’s last month of life aims to improve care through the identification of ineffective practices, enhance the quality of training provided to staff, and ensure the effective use of resources; all of which have the potential to change practice [10]. This study is significant since assessing the quality of care is an important activity for health care institutions to regularly undertake, yet there is a general recognition for the need to develop brief, quality measures for end-of-life care [14]. Also, gathering timely information is needed in order to monitor and improve the quality of end-of-life care, including valid and reliable data about the care provided, the recipients, the facilities, and the caregivers. By developing a brief chart audit tool that captures best practices derived from expert consensus and the research literature, NH facilities will be equipped with a valid means for monitoring and assessing the care delivered to residents in the last month of life. These assessments will help drive improvements in care by providing direction for staff education, the development of initiatives aimed at reducing ineffective practices, ensuring the optimal use of resources. These improvements will lead to a culture of care that aims to deliver the highest quality of care in the last phase of a resident’s life.

## Abbreviations

CNS: Clinical nurse specialist
NH: Nursing home
NP: Nurse practitioner
